# Root colonization by beneficial rhizobacteria

**DOI:** 10.1093/femsre/fuad066

**Published:** 2023-12-13

**Authors:** Yunpeng Liu, Zhihui Xu, Lin Chen, Weibing Xun, Xia Shu, Yu Chen, Xinli Sun, Zhengqi Wang, Yi Ren, Qirong Shen, Ruifu Zhang

**Affiliations:** State Key Laboratory of Efficient Utilization of Arid and Semi-Arid Arable Land in Northern China, The Institute of Agricultural Resources and Regional Planning, Chinese Academy of Agricultural Sciences, 12 Zhongguancun South Street, Beijing 100081, P.R. China; Jiangsu Provincial Key Lab for Organic Solid Waste Utilization, National Engineering Research Center for Organic-Based Fertilizers, Jiangsu Collaborative Innovation Center for Solid Organic Waste Resource Utilization, Nanjing Agricultural University, 6 Tongwei Road, Nanjing 210095, P.R. China; Experimental Center of Forestry in North China, Chinese Academy of Forestry, 1 Shuizha West Road, Beijing 102300, P.R. China; Jiangsu Provincial Key Lab for Organic Solid Waste Utilization, National Engineering Research Center for Organic-Based Fertilizers, Jiangsu Collaborative Innovation Center for Solid Organic Waste Resource Utilization, Nanjing Agricultural University, 6 Tongwei Road, Nanjing 210095, P.R. China; State Key Laboratory of Efficient Utilization of Arid and Semi-Arid Arable Land in Northern China, The Institute of Agricultural Resources and Regional Planning, Chinese Academy of Agricultural Sciences, 12 Zhongguancun South Street, Beijing 100081, P.R. China; State Key Laboratory of Agricultural Microbiology, College of Life Science and Technology, Huazhong Agricultural University, 1 Shizishan Street, Wuhan, P.R. China; Jiangsu Provincial Key Lab for Organic Solid Waste Utilization, National Engineering Research Center for Organic-Based Fertilizers, Jiangsu Collaborative Innovation Center for Solid Organic Waste Resource Utilization, Nanjing Agricultural University, 6 Tongwei Road, Nanjing 210095, P.R. China; Jiangsu Provincial Key Lab for Organic Solid Waste Utilization, National Engineering Research Center for Organic-Based Fertilizers, Jiangsu Collaborative Innovation Center for Solid Organic Waste Resource Utilization, Nanjing Agricultural University, 6 Tongwei Road, Nanjing 210095, P.R. China; Jiangsu Provincial Key Lab for Organic Solid Waste Utilization, National Engineering Research Center for Organic-Based Fertilizers, Jiangsu Collaborative Innovation Center for Solid Organic Waste Resource Utilization, Nanjing Agricultural University, 6 Tongwei Road, Nanjing 210095, P.R. China; Jiangsu Provincial Key Lab for Organic Solid Waste Utilization, National Engineering Research Center for Organic-Based Fertilizers, Jiangsu Collaborative Innovation Center for Solid Organic Waste Resource Utilization, Nanjing Agricultural University, 6 Tongwei Road, Nanjing 210095, P.R. China; Jiangsu Provincial Key Lab for Organic Solid Waste Utilization, National Engineering Research Center for Organic-Based Fertilizers, Jiangsu Collaborative Innovation Center for Solid Organic Waste Resource Utilization, Nanjing Agricultural University, 6 Tongwei Road, Nanjing 210095, P.R. China; State Key Laboratory of Efficient Utilization of Arid and Semi-Arid Arable Land in Northern China, The Institute of Agricultural Resources and Regional Planning, Chinese Academy of Agricultural Sciences, 12 Zhongguancun South Street, Beijing 100081, P.R. China; Jiangsu Provincial Key Lab for Organic Solid Waste Utilization, National Engineering Research Center for Organic-Based Fertilizers, Jiangsu Collaborative Innovation Center for Solid Organic Waste Resource Utilization, Nanjing Agricultural University, 6 Tongwei Road, Nanjing 210095, P.R. China

**Keywords:** rhizosphere, bacteria, root colonization, plant-microbe interactions, root exudates

## Abstract

Rhizosphere microbes play critical roles for plant’s growth and health. Among them, the beneficial rhizobacteria have the potential to be developed as the biofertilizer or bioinoculants for sustaining the agricultural development. The efficient rhizosphere colonization of these rhizobacteria is a prerequisite for exerting their plant beneficial functions, but the colonizing process and underlying mechanisms have not been thoroughly reviewed, especially for the nonsymbiotic beneficial rhizobacteria. This review systematically analyzed the root colonizing process of the nonsymbiotic rhizobacteria and compared it with that of the symbiotic and pathogenic bacteria. This review also highlighted the approaches to improve the root colonization efficiency and proposed to study the rhizobacterial colonization from a holistic perspective of the rhizosphere microbiome under more natural conditions.

## Teaser

We summarized the root colonizing process of the nonsymbiotic beneficial rhizobacteria, compared that with pathogenic and symbiotic rhizobacteria, and reviewed the approaches to improve the colonization efficiency.

## Introduction

The significance of plant- and animal-associated microbiomes to their hosts has been well recognized for decades (Mendes et al. 2013). Microbes inhabiting the rhizosphere are critical determinants of plant growth and health. Beneficial rhizobacteria show great potential in agricultural production since they offer a variety of beneficial functions for plants, such as promoting plant growth and enhancing plant abiotic stress tolerance by secreting phytohormones and some specific signaling molecules and protecting host plants by inducing systemic resistance and direct antagonism with soil-borne pathogens (Pieterse et al. 2014). These beneficial bacteria can generally be used in agriculture as biofertilizers or microbial agents and are essential in green agricultural production. Rhizosphere colonization is one of the most important features of rhizobacteria that determines their survival and propagation, which are prerequisites for versatile bacteria to exert their beneficial functions on host plants (Mendes et al. 2013).

**Figure 1.**
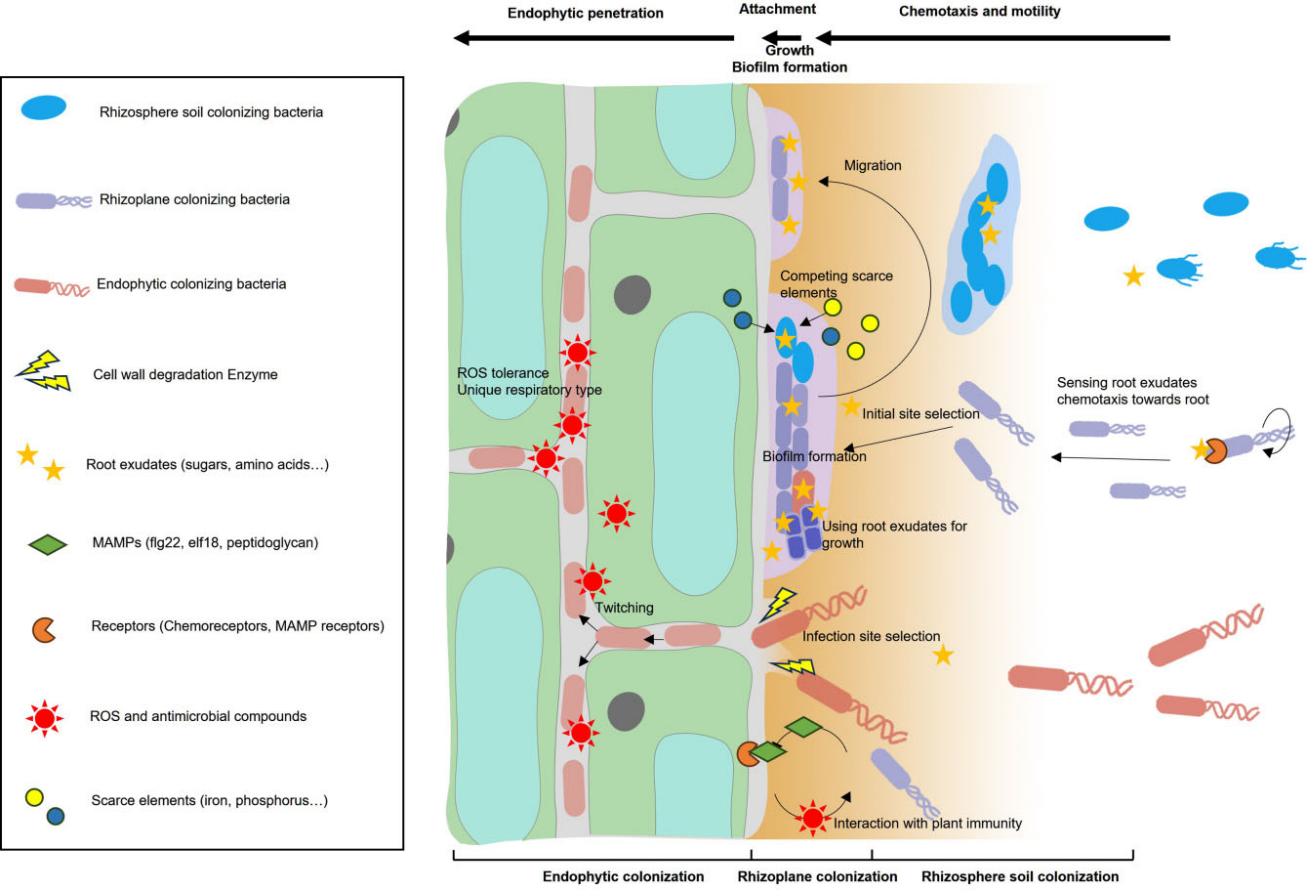
Rhizosphere colonization process of nonsymbiotic bacteria. Rhizosphere consists of the roots and the surrounding soil, and the rhizosphere colonization includes rhizosphere soil-, rhizoplane- and endophytic colonization. According to different bacterial species, the colonization process can be divided into several steps, including chemotaxis and motility, root surface attachment, growth and rhizoplane biofilm formation, and endophytic penetration. Chemotaxis and motility determine the moving toward rhizosphere, the initial site selection, and migration of colonization site. Attachment to the root surface is followed, during which the bacteria must overcome plant immunity. Bacterial growth using root exudates as the carbon resources and competing scarce elements in rhizosphere is necessary for biofilm formation, which is required by most rhizosphere soil and rhizoplane colonizing bacteria. Endophytic bacterial species penetrate intercellular spaces within root tissue through unique mechanisms after root attachment or biofilm formation.

The rhizosphere includes plant roots and the surrounding soil influenced by root exudates (Dessaux et al. 2016), therefore, bacteria surviving and forming firmly community in rhizosphere soil, on rhizoplane and in root endosphere were all defined as the term “rhizosphere colonization” (Fig. 1). They can selectively colonize distinctively on primary root or lateral root, on spatial axis of the root, inside root, or root surface. Rhizobacteria colonize the plant root in a highly heterogeneous manner, covering 10%–40% of the root surface (Danhorn and Fuqua 2007), and some endophytic bacteria can also live inside root tissue. Since the colonization process of symbiotic bacteria, which reside in living plant cells or is surrounded by a membrane compartment (Reinhold-Hurek and Hurek 2011), has been thoroughly reviewed (Roy et al. 2020, Soyano et al. 2021, Yang et al. 2022, González-Guerrero et al. 2023, Jain et al. 2023, Rahmat et al. 2023, Xu and Wang 2023), this review only focuses on the root colonization of nonsymbiotic beneficial rhizobacteria.

Plants are the major players in the rhizosphere and they affect bacterial colonization. Plants secrete 11%–40% of photosynthesis products into the rhizosphere as root exudates (Zhalnina et al. 2018, Du et al. 2021), which cause the rhizosphere to be a highly active site for microbial colonization than bulk soil. Undoubtedly, the colonization of beneficial rhizobacteria is largely impacted by the abundance and composition of root exudates. Root exudates can be divided into the low molecular weight and high molecular weight compounds. Low molecular weight compounds include sugars, organic acids, amino acids, alcohols, volatile compounds, and some secondary metabolites. The high molecular weight compounds are less diverse but yield a higher mass % of root exudates, and those compounds are mostly polysaccharides and proteins (Chagas et al. 2018). Although the rhizosphere is rich in carbon resources for bacterial growth, it is generally accepted that plants are able to expel unfavorable bacteria through the plant immune system, which is also a crucial factor that determines bacterial colonization in the rhizosphere (Shu et al. 2023). The concept of plant immunity has been well-established in interactions with pathogens and symbiotic microbes. Recently, the importance of plant immunity in modulating nonsymbiotic rhizobacteria colonization has been fully recognized (Shu et al. 2023). Additionally, a “cry-for-help” theory proposed that a stressed plant can recruit beneficial bacteria to colonize the rhizosphere (Lebeis et al. 2015, Rolfe et al. 2019). All these factors influence the rhizosphere colonization of the nonsymbiotic beneficial bacteria.

The biology of root colonization by rhizobacteria has advanced in recent years. Rhizosphere colonization is a complex process involving several steps that depend on bacterial lifestyles. They can colonize in rhizosphere soil, on rhizoplane, or endophytically based on some of these steps (Fig. 1). In general, rhizobacteria colonize the root in a sequential process that begins with rhizosphere chemotaxis, root attachment, sometimes followed by rhizoplane biofilm formation or endophytic colonization for some strains. Bacterial chemotactic motility involves a conserved intracellular signal transduction pathway and varied signal sensors and drives the selection of initial sites for attachment and colonization site migration, which vary depending on the strain and plant species (Sampedro et al. 2015, Li et al. 2022). After moving to the rhizosphere, some bacterial strains need to stop moving and adhere to the root surface, which is defined as root attachment (Knights et al. 2021). During this period, bacteria must exert their role to overcome the plant immune response for further colonization. Rapid proliferation using root exudates as the main carbon resources is one of most important process for colonization. Some of the rhizobacteria formed biofilm on the rhizoplane in a multispecies manner (Beauregard et al. 2013). During this period, bacteria have to compete for some scarce elements in the rhizosphere to support proliferation and biofilm formation (Liu et al. 2023). Additionally, some endophytes begin penetrating into plant tissue during life on the root surface (Dudeja et al. 2021, Mushtaq et al. 2023). In general, these processes involve complicated lifestyle transformation and intracellular signal transduction that are influenced by plants and the environment. However, the current understanding of bacterial colonization in the rhizosphere is scattered, especially for beneficial nonsymbiotic rhizobacteria.

In this review, we will summarize the knowledge on the rhizosphere colonization of nonsymbiotic beneficial bacteria along with the sequential process and conclude the underlying regulatory molecular mechanism, the important bacterial genes involved in the processes, and the influencing factors. We will also review the advances in “cry-for-help” theory. The difference in colonization processes and the plant‒microbe interactions that determine colonization between nonsymbiotic bacteria will be compared with that of symbiotic/pathogenic bacteria. Finally, we propose several artificial strategies to enhance the colonization of beneficial rhizobacteria, which would benefit the application of beneficial rhizobacteria in agriculture. The scope of this review is comprehensively summarizing the rhizosphere colonization processes of the nonsymbiotic bacteria to promote the application of beneficial rhizobacteria in agriculture.

## Chemotaxis and motility

Chemotaxis is a motility-based ability of microbes to sense chemical gradients and direct their movement either up the gradient toward the source (attraction) or down the gradient away from the source (repulsion). Motility and chemotaxis of vegetative bacterial cells are essential for rhizosphere colonization, as well as for establishing primary bacteria–root interactions (Feng et al. 2021a). Root exudates activate chemosensory pathways and cause motile bacteria to move toward the root. Rhizobacterial motility can be achieved by various mechanisms, including flagellar swimming, swarming, twitching, and gliding motility (Kearns 2010). Bacterial swimming is achieved by rotating flagella to generate a force that moves the cell forward (Sampedro et al. 2015). Swarming is a multicellular movement over a solid surface that is driven by a raft-like flagellar complex from the community (Kearns 2010). Twitching is a motility based on the extension–tethering–retraction–extension of type IV pili (Sampedro et al. 2015). Gliding motility is a definition of cells moving smoothly along their long axis in the absence of any visible organelle (Mignot 2007).

Chemotaxis and motility then drive the selection of the initial contact site on the root. The success of these processes determines the root colonization efficiency. It is evident that either inactivation of chemosensory activity by knocking out all the chemotaxis receptors or blocking motility by deleting the genes responsible for synthesizing flagellin in a rhizobacterium led to a 100-fold decrease in root colonization efficiency (Feng et al. 2018, Tzipilevich et al. 2021).

### Chemotaxis process and signaling

Chemotaxis intracellular signaling is conserved in many bacterial species. Bacterial chemotaxis toward root exudates is initiated by the perception of chemoeffectors in root exudates by bacterial transmembrane chemotaxis receptors, which are specifically termed methyl-accepting chemotaxis proteins (MCPs) (Feng et al. 2021a). Generally, chemotaxis receptor proteins always exist in a ternary complex with the CheA histidine kinase and the coupling protein CheW. Chemotaxis receptors are transmembrane proteins that constitute a highly varied ligand-binding domain (LBD) in the extracellular space for signal sensing, an intracellular highly conserved methyl-accepting (MA) domain for adaptation, which is the standard criterion for the annotation of proteins as MCPs (Sampedro et al. 2015). The MCPs selectively recognize and bind to specific ligands, such as root exudates, resulting in molecular signals that transduce across the cellular membrane. This transduction subsequently modulates the autophosphorylation rate of the histidine kinase CheA in a CheW-dependent manner (Lacal et al. 2010). CheA and CheY constitute a two-component system. The phosphorylation of CheA affects the transphosphorylation of the CheY response regulator. Phosphorylated CheY binds to motor proteins that are responsible for driving various kinds of motility in different bacteria. In addition, the turnover of methylation and demethylation of the MA domain of the MCPs was deployed as an adaptation system, and methylation increased while demethylation decreased the autophosphorylation activity of CheA (Sampedro et al. 2015). This whole signaling pathway is extremely well-conserved in many bacteria, including *Escherichia coli, Bacillus* spp. and *Pseudomonas* spp.

The variety of MCPs with different LBDs determines the molecules to be sensed by the bacteria (Sanchis-López et al. 2021). In rhizobacteria, an expansive array of MCPs and their corresponding ligands have been identified, with notable examples found in species such as *Pseudomonas putida, Bacillus velezensis*, and *Sinorhizobium meliloti*. Allard-Massicotte et al. (2016) demonstrated that root colonization of *Bacillus subtilis* involves multiple chemotaxis receptors. An efficient colonizer in the rhizosphere should respond to a broad range of compounds in root exudates. For example, the colonization of *P. putida* KT2440 and *B. velezensis* SQR9 was regulated by various compounds in root exudates (Ortega et al. 2017, Feng et al. 2019). Notably, *Pseudomonas* spp. exhibit chemotactic responses to an impressive repertoire of over 140 compounds, thereby setting them as exemplary models for elucidating the structure‒function relationships between MCPs (Sampedro et al. 2015). A comprehensive analysis revealed that *P. putida* KT2440 harbors as many as 27 distinct MCPs (Corral-Lugo et al. 2016), each specific to detect a myriad of signaling molecules, including polyamines, amino acids, fatty acids, sugars, and many secondary metabolites. *Bacillus velezensis* SQR9 is endowed with eight unique MCPs, explicitly enumerated as McpA, McpB, McpC, McpR, TlpA, TlpB, YfmS, and HemAT (Liu et al. 2020b). However, the functions of homologous MCPs can be different between strains. For example, McpA in *B. velezensis* SQR9 orchestrates chemoattraction to a wide range of 20 ligands, including organic acids, sugars, and amino acids (Feng et al. 2019). Its homologs in *B. subtilis* NCIB 3610 are predominantly predisposed to sugar ligands, specifically glucose and α-methylglucoside (Allard-Massicotte et al. 2016). Through rigorous molecular investigations coupled with site-directed mutagenesis experiments, it has been elucidated that McpA in strain SQR9 boasts a broad ligand-sensing capacity arising from its capability to harness both the distal and proximal membrane regions of its LBD. (Feng et al. 2022). Root-secreted glucose can act as a chemoattractant to many beneficial rhizobacteria (Feng et al. 2019, Sánchez-Gil et al. 2023). Cucumber root-secreted d-galactose serves as a ligand of McpA in strain SQR9 to enhance chemotaxis (Liu et al. 2020b). Compounds that act as chemoeffectors in root exudates are mainly low molecular weight compounds, such as organic acids, amino acids, sugars, sugar alcohols, and flavonoids. Some of these compounds also act as repellents. Detailed MCPs and their sensed root exudate compounds have been summarized by Feng et al. (2021a).

In addition to acting as a chemoeffector attracting bacteria, a range of compounds in root exudates enhance the motility of rhizobacteria. Root-secreted sucrose activates the bacterial production of extracellular polymeric levan, which in turn regulates the flagellar synthesis of *B. subtilis*, and *B. subtilis* cannot effectively colonize roots of *Arabidopsis* mutants that are deficient in root sucrose secretion (Tian et al. 2021b). Interestingly, *Bacillus*-produced surfactin, an antibiotic essential for bacterial motility and thus rhizosphere colonization, is also promoted by other root exudates, such as polysaccharides (Debois et al. 2015, Hoff et al. 2021). Recent studies revealed that root-secreted inositol can act as a signaling molecule to stimulate swimming motility in *Pseudomonas* via inositol-induced repression of DksA, a transcriptional regulator involved in inhibiting swimming motility and thus chemotaxis to the rhizosphere (Vílchez et al. 2020, O’Banion et al. 2023, Sánchez-Gil et al. 2023). The *Arabidopsis* root-secreted flavonoids attract *Aeromonas* sp. H1 by upregulating transcripts of flagellum biogenesis and inhibiting fumarate reduction for smooth swims (He et al. 2022).

Notably, the diffusion range of root exudates is inherently limited, leading to reduced concentrations at greater distances from the root. In light of emerging theories on bacterial chemotaxis, there appears to be a sophisticated relay of chemotactic signals between distinct bacterial cells (Cremer et al. 2019, Insall et al. 2022). Although they have not identified the signaling molecules secreted by the bacteria yet (Cremer et al. 2019), it supports that bacterial self-generated chemotactic signals might be essential in facilitating movement to the rhizosphere. Besides by sensing self-produced signal, bacterial chemotaxis may also be achieved through microbe–microbe interactions (Tian et al. 2021a), sometimes even by attraction to the exudates of root-associated fungi (Jiang et al. 2021, Mesny et al. 2023). To encapsulate this dynamic, microbes near the roots will sense root-secreted chemotactic signals and secrete chemotactic cues from their locus. This results in the establishment of a secondary chemotactic signal gradient, effectively drawing in more bacterial cells and mediating bacterial advancement toward the roots.

### Colonization site selection and migration

Bacterial chemotaxis and motility determine colonization site selection and migration. The colonization sites can differ between bacteria, even between phylogenetically close strains (Fan et al. 2012, Gao et al. 2013, Tovi et al. 2019, O’Neal et al. 2020). It can be expected that sites with high exudation are possible colonization hotspots for the whole community because the high concentration of root exudates would attract bacteria (Darrah 1991, Marschner et al. 2011). Root hairs promote plants to allocate more carbon to root exudates (Holz et al. 2018), but it is generally agreed that the exudation rates are high in the elongation zone just behind the root tips rather than in the mature root zones. The colonization site is temporally changed along the root axis or between different root branches during the colonizing life cycle (Trivedi et al. 2020). The long-term colonization site may be different from the initial contact site. For instance, *Bacillus megaterium* NCT-2 cells were mostly distributed in the epidermis of the root elongation zone of maize at 3 days postinoculation (dpi), while colonization was observed along the meristematic zone, elongation zone, and root hair region at 11 dpi (Chu et al. 2018).

First, bacterial chemotaxis and motility contribute decisively to the selection of the initial site for colonization. O’Neal et al. (2020) found that the *Azospirillum brasilense* mutant lacking the major chemoreceptors that are responsible for root exudate chemotaxis is impaired in preferentially accumulating on rhizoplane and inside tissue of maturation and elongation zones. The factors influencing bacterial chemotaxis and motility for selecting root colonization sites are complex, including the diversity and concentration of each component in root exudates at different sites and the immune response of different cell types and some locally secreted antimicrobial compounds (Fröschel et al. 2021, Verbon et al. 2023). For example, reactive oxygen species (ROS) produced by roots also act as repellents to drive bacterial repulsion from the wheat root tip for initial colonization (O’Neal et al. 2020). Second, in addition to having a decisive role in the initial contact site, bacterial chemotaxis and motility also drive colonization site migration after root surface attachment. Root cell development changes the root exudation site, and bacterial migration could follow the changed root exudation sites, which are dynamically moving, following the expansion of the root system (Zboralski and Filion 2020). The migration of bacterial colonization site on roots after initial colonization can also result from evasion of immune-activating sites. Spatiotemporal root immune responses during microbial colonization are an important factor that determines the bacterial colonization site (Tsai et al. 2023). Liu et al. (2018) suggested that the ΔmorA mutant of *Pseudomonas* is a poor rhizosphere colonizer due to its inability to move from the initial site of colonization after triggering plant immune responses, indicating that migration along the root may occur to evade plant defense after initial colonization.

Overall, there is no doubt that bacterial chemotaxis and motility determine the site preferences for colonization in different root zones. However, most of the current research measuring rhizosphere colonization is mainly based on overall quantitative measurements, while measurements of colonization in different root zones are lacking, which will lead to many objectively existing differences in colonization being ignored or some differences in colonization being misinterpreted. The key problem for this status is the difficulty in measuring bacterial colonization within distinct root zones. Most current studies regarding colonization sites are based on microscopic observations, including fluorescence-, GUS- and FISH-based methods (Cao et al. 2023b). These strategies can well-reflect *in situ* bacterial colonization, but they are not as accurate as traditional plate counting methods in quantification. Moreover, due to the limitation of displaying only partial root zones under the microscope, it can sometimes be influenced by subjective bias.

## Root surface attachment and interaction with plant immunity

Root surface colonization begins immediately after chemotaxis toward root, with successful adhesion to the root being the critical step for rhizoplane and endophytic colonization. In brief, bacteria need to stop moving and bind to the root surface, in which a transformation of lifestyle processes controlled by complex signal transduction is involved. Comprehensive studies on representative rhizobacteria, including *Pseudomonas, Bacillus, Bradyrhizobium, Azospirillum, Agrobacterium*, and *Salmonella*, have unveiled the molecular intricacies of root attachment. It has been established that agriculturally important microbial species share a biphasic model for root attachment (Wheatley and Poole 2018, Knights et al. 2021). In most cases, this biphasic model involves two steps: initial attachment occurs when rhizobacteria are reversibly bound to a root surface, followed by secondary attachment that results in their irreversible attachment (Knights et al. 2021). The current knowledge on root attachment based on these two steps will be reviewed here. In addition, upon attachment to the root surface, plant immunity functions as an important factor influencing bacterial colonization, which will also be discussed for the strategies bacteria deployed to address plant immunity.

### Root surface attachment process

#### Reversible initial attachment

In general, initial attachment is weak, reversible, and nonspecific, allowing single cells to attach to the root surface. Compared to later-stage secondary attachment, the initial attachment is relatively poorly characterized. Numerous physiochemical and electrostatic forces influence the initial interactions between the surface molecules of the root and bacterial cell envelope, including van der Waals forces, electrostatic forces, and hydrophobic interactions. To overcome these repulsive forces, rhizobacteria use adhesive pili (T-pili), flagella, the polar flagellum, and fimbriae to overcome the electrostatic repulsion that occurs between negatively charged cell envelopes and root surfaces (Berne et al. 2015, Knights et al. 2021). For instance, the flagella-deficient mutant of *A. brasilense* is unable to adhere to wheat or maize roots. Moreover, the polar flagella purified from *A. brasilense* bind to wheat roots directly (Rossi et al. 2016). In addition to this universal force of attachment, rhizobacteria can exhibit numerous species-specific mechanisms for attachment and colonization. The major membrane porin, outer membrane proteins, and polysaccharides are considered to play a role in root adhesion during the early stages of root establishment (Berne et al. 2015). An outer membrane porin F (OprF) from *Pseudomonas* shows adhesive properties toward the roots of cucumbers and tomatoes. It was found that OprF-deficient mutants of *P. fluorescens* are significantly less capable of loosely adhering to roots than wild-type plants, which indicates that OprF plays an important role in primary attachment (Alvarez Crespo and Valverde 2009). Although OprF in *Pseudomonas* appears to play a role in initial attachment, its molecular mechanism remains unclear.

#### Irreversible secondary attachment

In the following stages of initial bacterial attachment, only a small percentage of rhizobacteria switch to a stronger, more specific binding mode and generate extracellular fibrils that facilitate bacterial accumulation and aggregation, called secondary attachment (Wheatley and Poole 2018). A range of species-specific strategies are employed by rhizobacteria for secondary attachment. *Pseudomonas* spp. secrete a Ca^2+^-binding protein, LapA, via ATP-binding cassette transporters. This protein loosely associates with bacterial surfaces, facilitating interactions with the root surface (Hinsa et al. 2003). LapA of *P. putida* is also necessary for attachment to abiotic surfaces and to plant seeds (Espinosa-Urgel et al. 2000). Notably, *P. fluorescens* mutants lacking LapA exhibit diminished initial attachment to abiotic surfaces and compromised biofilm formation abilities. The O-antigenic chains of *Pseudomonas* spp. lipopolysaccharides have also been linked to root attachment in crops such as tomatoes and potatoes (Spiers and Rainey 2005). Zhao et al. (2016) demonstrated that collagen-like proteins of *B. velezensis* FZB42 are critical for root attachment. Recently, Huang et al. (2022) demonstrated that the wall teichoic acid, flagellar protein FliD, and YhaN (a putative ABC transporter) of *B. velezensis* SQR9 function as adhesins on both cucumber root surfaces and abiotic surfaces and are involved in rhizosphere colonization (Huang et al. 2022). Cyclic di-AMP, a common bacterial second messenger, influences the formation of biofilms and plant root attachments in *B. subtilis* (Townsley et al. 2018). These investigations underscore that root attachment mechanisms are pivotal for successful rhizosphere colonization by bacteria.

### Interaction with plant immunity

Plant immunity is one of the barriers that rhizobacteria must overcome during attachment to the root surface. The first process depends on recognizing highly conserved microbe-associated molecular patterns (MAMPs), including flg22, chitin, peptidoglycan, and lipopolysaccharide, by pattern recognition receptors (PRRs) and activating pattern-triggered immunity (PTI), which forms a primary defense against microbial colonization. The second layer of plant immunity is referred to as effector-triggered immunity. Plants have evolved nucleotide binding and oligomerization domain-like receptors, which sense microbial effectors either directly or through effector-induced modifications of host structures (Wang et al. 2022b). H^+^/Ca^2+^ ion fluxes and bursts of ROS are two typical cellular responses occurring within minutes after immune signaling responses. Other responses include triggering downstream defense-related gene activation, defense hormone regulation, callose deposition, camalexin biosynthesis, and antimicrobial compound accumulation. This local immune response is always accompanied by growth inhibition as a result of the growth-defense trade-off (Liu et al. 2013). In addition to triggering the local immune response, beneficial rhizobacteria can also elicit the induction of systemic resistance (ISR) (Pieterse et al. 2014).

Evidence show that at least the PTI is engaged and influences root colonization by beneficial rhizobacteria (Yu et al. 2019b). A recent study demonstrated that the *Arabidopsis* root bacterial community is involved in PTI regulation, and a group of robust, taxonomically diverse PTI-inhibiting strains that are efficient root colonizers were identified (Teixeira et al. 2021). In addition to facilitating the colonization of PTI-regulating bacteria themselves, both individual strains and synthetic consortia that regulate PTI can increase the ability of other beneficial bacteria to colonize roots (Ma et al. 2021, Teixeira et al. 2021). This suggests that the interaction with plant immunity highly influences the root colonization of beneficial rhizobacteria.

#### Suppressing the root immune response

Increasing evidence demonstrates that beneficial rhizobacteria can avoid being detected by root receptors that elicit immune responses, which are negative for bacterial colonization and plant growth. One aspect is the variation in the MAMPs, which is evidenced by the variation in flg22, one of the well-studied MAMPs. Colaianni et al. (2021) showed that most of the flg22 peptide variants from beneficial bacteria failed to activate PRR FLS2 (64%) and did not significantly inhibit plant host growth (80%), suggesting no activation of an energy-costly immune response. This kind of flg22 peptide variant altered PTI signaling output by interfering with coreceptor enlistment and by another, unidentified mechanism that triggered the typical ROS response, resulting in modulation of plant immunity (Colaianni et al. 2021). This finding suggests that beneficial rhizobacteria may avoid eliciting the root immune response by deploying flagella with low immunogenic sequences to facilitate rhizosphere colonization. The advantages of a low-immune-response-eliciting flagellin also drive the evolution of bacterial flagellar sequences with a trade-off of motility (Parys et al. 2021). In addition, there are beneficial rhizobacteria that possess immunogenic MAMPs that are very similar to those of pathogens. They have, therefore, evolved the ability to evade PRR recognition by inhibiting the interaction of their MAMP with PRRs, including through modification of the MAMP epitope, inhibition of the biosynthesis of MAMP-containing molecules, or alteration of microbial cell wall compositions (Yu et al. 2019b). In contrast to the phytopathogen *Pseudomonas syringae*, which suppresses the root immune response by producing the low molecular weight phytotoxin COR, the beneficial rhizobacterium *Pseudomonas* suppresses the flg22-triggered immune response without producing COR (Millet et al. 2010). Instead, Yu et al. (2019a) demonstrated that *Pseudomonas capeferrum* WCS358 reduces the rhizosphere pH by producing gluconic acid and its derivative 2-keto gluconic acid, therefore inhibiting the flg22-binding activity of FLS2, which requires a neutral pH environment. The inhibition of FLS2 activity further suppresses the flg22-mediated oxidative burst and root immunity, thereby facilitating colonization (Yu et al. 2019a). Similarly, the beneficial *B. subtilis* FB17 can suppress flg22-induced early root immune responses in *Arabidopsis* by releasing an unidentified low molecular weight compound, which controls the JA signaling components JAR1, JIN1, and MYC2 (Lakshmanan et al. 2012). This suggests that beneficial rhizobacteria actively interfere with plant immune signaling by delivering immune-suppressive compounds. However, current knowledge on suppressing PTI is mainly aimed at flg22, and more efforts aimed at other MAMPs on a large scale should be made to reveal immune suppression by beneficial rhizobacteria during colonization.

#### Tolerance of root immune response

Once plant immunity is activated, some beneficial rhizobacteria can also utilize strategies to address the activated immune response. The root cell-type-specific transcriptome in response to a beneficial rhizobacterium *Pseudomonas simiae* WCS417 revealed a spatial difference in immune activation of root hairs, cortex and endodermal barrier during colonization of this strain, suggesting that a spatial selection of the colonization site would benefit immune response evasion (Verbon et al. 2023). A genome-wide screen in rhizosphere *Pseudomonas* identified two genes, morA and spuC, that are essential in rhizosphere colonization, and the authors speculated that these two genes may confer the bacterium an ability to disperse from the initial site of colonization after triggering plant immune responses (Liu et al. 2018). This case proposed a potential bacterial strategy that evades root immunity through spatial mitigation of the colonization site. In addition to spatial evasion, higher tolerance is another strategy to address the activated root immune response, such as the ROS burst. Recently, Song et al. (2021) demonstrated that ROS in roots regulate the levels of rhizosphere beneficial *Pseudomonas*. The auxin produced by the beneficial bacterium *B. velezensis* FZB42 is essential for root colonization by antagonizing ROS produced as part of the receptor EFR-triggered immune response (Tzipilevich et al. 2021). Moreover, ROS induce auxin synthesis in *B. velezensis* FZB42 (Tzipilevich et al. 2021). The beneficial rhizobacterium *B. velezensis* SQR9 possesses a specific two-component regulatory system (TCS), ResDE, to tolerate the ROS produced during the flg22-triggered root immune response, which promotes rhizosphere colonization of this strain (Zhang et al. 2021).

However, it is still unclear whether the suppression of PTI in roots by beneficial rhizobacteria increases the risk of root infection by soil-borne pathogens. From the results reported by Ma et al. (2021), it seems that suppression of root PTI by beneficial rhizobacteria renders plants more susceptible to opportunistic *Pseudomonas* pathogens. Moreover, beneficial rhizobacteria can stimulate ISR, but the plant immune system actively or passively overlooks colonization by beneficial rhizobacteria during interactions. Whether this resistance impacts the colonization of nonsymbiotic beneficial rhizobacteria and its relationship with local plant immunity is unclear.

## Bacterial growth and biofilm formation

In the rhizosphere, bacterial growth using root exudates as carbon resources is an important factor influencing root colonization. In addition to carbon resources, some scarce elements, such as phosphorus and iron, are also factors limiting the colonization of bacteria. Many bacterial species have evolved fascinating strategies to compete for scarce elements. Moreover, biofilm formation is an important process for many rhizoplane-colonizing bacterial species, motile flagellated bacterial cells differentiate into matrix-producing cells, which stop agglutinating, begin and form extracellular matrix surrounding chains (Karygianni et al. 2020). The biofilm matrix binds cells and imparts many key features to the biofilm, and therefore rhizosphere colonization (Flemming et al. 2023). The biofilms in rhizosphere are generally formed by bacteria from multispecies, and the matrix provides a spatial structure and multiple levels of protection for the community within biofilm.

### Bacterial growth using root exudates

Bacterial growth and nutrition are the most important factors influencing bacterial colonization in the rhizosphere (López et al. 2023), and root exudate compounds can serve as nutrients that support bacterial colonization. The ability to utilize nutrients in root exudates is critical for rhizobacteria to occupy rhizosphere niches. Sugars and organic acids constitute a large fraction of exudates and are the major carbon sources for rhizobacteria (Sasse et al. 2018, Korenblum et al. 2022); some root-sourced VOCs, such as terpenes, can also act as nutrient sources (Schulz-Bohm et al. 2018). Plant root exudate nutrients can selectively promote the colonization of specific bacteria (Wang et al. 2022a). For instance, Huang et al. (2019) discovered that the specialized triterpenes thalianin, thalianyl fatty acid esters, and arabidin in root exudates of *Arabidopsis* modulate the root microbiota by enhancing or inhibiting specific bacterial growth. Rhizobacteria that can selectively metabolize certain triterpenes as carbon sources for growth have more efficient rhizosphere colonization. The root-secreted compound 1-aminocyclopropane-1-carboxylic acid (ACC), which is the precursor of ethylene, can be used only by bacteria with ACC deaminase. These bacteria can degrade ACC as a nitrogen source, giving them a significant advantage in rhizosphere colonization (Li et al. 2019). Recently, several publications demonstrated that plant secreted inositol as a nutrient is important for regulating rhizobacteria colonization (O’Banion et al. 2023), and a conserved inositol metabolism cluster in root *Pseudomonas* contributes to the competition for nutrients in the rhizosphere (Sánchez-Gil et al. 2023). In addition to the direct effect, compounds in root exudates can be degraded by specific bacteria, and the resulting metabolites will promote colonization by other bacteria. This kind of effect is expected to greatly participate in modulating root colonization by beneficial rhizobacteria (Sasse et al. 2018).

Some broad-spectrum antimicrobial substances in root exudates also impact the colonization of beneficial rhizobacteria by serving as carbon resources. Many plant secondary metabolites and small peptides exert variable antimicrobial activity (Chagas et al. 2018) and function as bioprotectants against pathogens. However, some of these compounds have selective antimicrobial activity and can act as carbon resources for certain beneficial rhizobacteria. Rhizobacteria that can metabolize root-secreted antimicrobial substances will have higher rhizosphere colonization efficiency and succeed in root colonization. The root-secreted toxic compounds camalexin and benzoxazinoids, which are signatures of the root immune response, also promoted colonization by beneficial *Pseudomonas* (Hu et al. 2018, Koprivova et al. 2019). Many VOCs produced by roots can serve as antimicrobial compounds, such as terpenes and terpenoids, to inhibit pathogen growth, and interestingly, they can also promote specific beneficial rhizobacterial growth (Chagas et al. 2018, Schulz-Bohm et al. 2018). In addition, aromatic compounds released by roots also mediate defense mechanisms against pathogens and attract some microbes by serving as carbon sources (Lattanzio et al. 2006). Indeed, Lebeis et al. (2015) demonstrated that salicylic acid, an aromatic signaling molecule responsible for many kind of plant defense response, can be used by some beneficial bacterial strains as a growth signal or as a carbon source.

Some specific transporters from either plants or bacteria have been suggested to be involved in the process of bacterial acquisition of root secreted carbon resource and contribute to the bacterial colonization in rhizosphere. Plants have developed active mechanisms for root exudation. Numerous studies have established that specific transporters located on the plasma membrane of root may be responsible for recruiting beneficial bacteria (Hennion et al. 2019, Vives-Peris et al. 2020). The plant transporter ALMT1 plays a role in exudation of the malate and the gamma-aminobutyric acid (GABA), which is one of the major carbon resources for rhizobacteria (Lakshmanan et al. 2012, 2013, Kamran et al. 2020). *Arabidopsis* amino acid transporter, LHT1, modulates *P. simiae* metabolism in the rhizosphere, which influence its colonization efficiency (Agorsor et al. 2023). Bacterial also deploy a range of transporters to acquire the root exudates. Using a combination of comparative genomics and exometabolomics, Zhalnina et al. (2018) revealed that the uptake of root-secreted carbon resources by specific transporters of rhizobacteria determines their colonization, and a bacterium with an uptake transporter of the highly abundant nutritional compounds of root exudates will be highly advantageous in rhizosphere colonization. They also found that the uptake of certain substances is highly variable among rhizobacteria (Zhalnina et al. 2018). Under controlled conditions, Lin et al. (2020) demonstrated that knockout of the *ptsG* gene encoding the main glucose transporter in *Bacillus cereus* C1 L led to a sharp decrease in root colonization, suggesting the importance of bacterial transporter of root secreted carbon resources in bacterial colonization.

### Biofilm formation

The formation of a biofilm is a way to maintain a critical cell mass in a specific location that is sufficient to initiate beneficial interactions with host plants (Flemming and Wuertz 2019). Biofilms increase resistance to certain environmental stresses as well as antimicrobial tolerance, protection from protozoan predation, consortia metabolism, or the opportunity for horizontal gene transfer (Arnaouteli et al. 2021). The biofilm matrix consists of extracellular polymeric substances, including polysaccharides, proteins, amyloids, lipids, and extracellular DNA, as well as membrane vesicles and humic-like refractories (Flemming et al. 2023).

#### Global transcription factors in biofilm formation

Mature biofilm formation generally indicates successful rhizosphere colonization. Rhizobacterial biofilm formation on the root surface is a highly regulated process, as each species has its own molecular mechanism for responding to environmental cues (Trivedi et al. 2020). The cessation of movement and initiation of biofilm formation by beneficial rhizobacteria are typically governed by one or several global transcriptional regulators within the bacterium. Consequently, these two cellular decisions are always coupled. When cells opt to transition into a biofilm state, the gene transcription associated with motility and chemotaxis is simultaneously downregulated. For example, biofilm formation by beneficial *Bacillus* in rhizosphere is governed by two global transcription factors, Spo0A and DegU (Arnaouteli et al. 2016, Kobayashi and Ikemoto 2019). DegU controls both motility and biofilm formation by different phosphorylation levels (Kobayashi and Ikemoto 2019). Spo0A also controls sporulation and biofilm formation by different phosphorylation levels (Xu et al. 2019a). *Pseudomonas* deploys different oligomerization of the global transcriptional regulator FleQ to adjudge the decision of motility and biofilm formation (Nie et al. 2022). Deficiency of these global transcriptional regulators in bacteria always leads to sharply reduced rhizosphere colonization (Xu et al. 2014, 2017, Emonet et al. 2021), suggesting the critical role of lifestyle transitions in rhizosphere colonization. Such a mechanism will prevent the contradictory coactivation of biofilm formation and motility during rhizosphere colonization.

The global transcriptional regulators that direct the shift from bacterial motility to biofilm formation respond to environmental cues, such as root exudates (Ivanova et al. 2023). This sensory mechanism is generally mediated by cell surface receptors such as histidine kinases, notably KinD in *Bacillus* (Liu et al. 2020a). Upon perceiving specific rhizosphere signals, these receptors communicate with global regulatory factors in various ways depending on bacterial variations (Arnaouteli et al. 2021, Nie et al. 2022, Wang et al. 2022), prompting cells to initiate biofilm formation on root surfaces. Certain plant polysaccharides, the major components of the plant cell wall, were also shown to enhance the biofilm of *B. subtilis* by acting as signals for controlling the phosphorylation level of the master regulator Spo0A and as carbon resources for producing the matrix exopolysaccharide (Beauregard et al. 2013). Interestingly, some signaling molecules induce both biofilm formation and trigger chemotaxis in beneficial rhizobacteria, such as cucumber root-secreted d-galactose, which could be induced by *B. velezensis* SQR9, serving as a signal for enhancing chemotaxis and biofilm formation of strain SQR9 in a McpA-dependent manner (Liu et al. 2020b). The organic acids in the root exudates of peanut, including citric, malic, and oxalic acids, promoted bacterial biofilm formation of the beneficial rhizobacterium *Burkholderia pyrrocinia* strain P10 in rhizosphere (Han et al. 2023). In addition, the flavones in rice root exudates enhance biofilm formation of the nitrogen-fixing bacterium *Gluconacetobacter diazotrophicus*, and biofilm formation in turn recruits diazotrophic bacteria in the rhizosphere (Yan et al. 2022). While these are distinct processes in rhizosphere colonization, it can be expected that bacteria might exhibit differential responses to different concentrations of the same signaling molecule. Thus, a molecule could stimulate chemotaxis at greater distances from roots but favor biofilm formation on the root surface. Such dose-dependent signaling is very common in biofilm and chemotaxis regulation among rhizobacteria.

#### Effect of self-produced secondary metabolites on biofilm formation

Rhizosphere microorganisms can produce many secondary metabolites, which also impact biofilm formation. Root-secreted sucrose activates the bacterial production of extracellular polymeric levan, which in turn regulates the synthesis of surfactin and hyperflagellation of the bacterium (Tian et al. 2021b). Interestingly, by causing potassium leakage, surfactin was demonstrated to be an essential signaling molecule in the establishment of biofilms and root colonization in *B. subtilis* NCIB3610 (Lopez et al. 2009). It has also been shown that another lipopeptide antibiotic, bacillomycin D, contributes to biofilm formation by facilitating iron acquisition. In *B. velezensis* SQR9, bacillomycin D specifically promotes transcription of the iron ABC transporter FeuABC by binding to its transcription factor, called Btr (Xu et al. 2019a). Additionally, using a novel branched-chain fatty acid, bacillunoic acid, allows *B. velezensis* SQR9 to utilize a novel branched-chain fatty acid called bacillunoic acid to establish a policing system for punishing cheaters within the biofilm community and to improve the community’s fitness in a variety of conditions, including the root colonization process (Huang et al. 2023). Importantly, numerous studies have observed that siderophores play an important role in rhizobacterial biofilm formation of *Bacillus* spp. and *Pseudomonas* spp. siderophore-defective mutants in different PGPR strains fail to form biofilms and are unable to competitively colonize plant roots (Pizarro-Tobías et al. 2015, Qin et al. 2019, Singh et al. 2022a). Owing to the complexity of secondary metabolites in the rhizosphere, there are numerous secondary metabolites that affect the interaction between plants and rhizobacteria, which needs to be investigated further.

#### Multispecies biofilm in the rhizosphere

It has been recognized that multispecies biofilms, rather than single-species biofilms, are the most dominant bacterial lifestyle naturally found in the rhizosphere, a consortium of bacterial isolates may form stronger biofilm on rhizoplane thus an enhanced colonization can be expected (Burmølle et al. 2014, Sadiq et al. 2021). There have been numerous recent studies that provide insight into the synergistic effects of multispecies biofilms in rhizosphere soil, resulting in beneficial properties for plants. For example, a four-species biofilm consortium exhibited higher biomass than single species, as well as increased tolerance to environmental stress (Ren et al. 2015, Yang et al. 2021). In one particular instance, a consortium of five rhizosphere native bacterial isolates forms synergistic biofilms in vitro and colonizes a larger area on the root than the individual strains (Santhanam et al. 2015, 2019). Inoculation of cucumber rhizosphere with *B. velezensis* could increase the colonization of resident plant-beneficial *Pseudomonas stutzeri* through synergic biofilm formation (Sun et al. 2022). Furthermore, a study demonstrated that a three-species combination composed of *Xanthomonas, Stenotrophomonas*, and *Microbacterium* spp. showed increasing biofilm production compared to their individual members and thus increasing beneficial function on *Arabidopsis* (Berendsen et al. 2018).

### Competition for scarce elements for growth and biofilm formation

Because of the large number of organisms in the rhizosphere, there are inevitable wars for limited elements, especially for the relatively scarce nutrient elements that are essential for rhizobacterial colonization, such as phosphorus, iron, zinc, and manganese (Dennis et al. 2010, Tsai and Schmidt 2017). Here, the scarce element nutrient is defined as the limited amount of this element in the rhizosphere becomes a limiting factor for bacterial growth and biofilm formation. In addition, plants also need these elements for growth, leading to fierce competition for phosphorus and iron in the rhizosphere.

Phosphorus generally reacts with calcium and magnesium in alkaline soils or with aluminum and iron in acidic soils to be fixed, which is difficult to absorb and utilize, resulting in a low level of phosphorus availability for bacteria (Earth System Science Data Discussions 2017). Rapid root absorption and poor mobility often lead to phosphorus depletion in the rhizosphere (Ceulemans et al. 2017, Sakuraba et al. 2018). Soil phosphorus is divided into inorganic P (P_i_) and organic P (P_o_); inorganic phosphorus mainly exists in the form of phosphate, and organic P is an insoluble complex formed with organic monoesters, diesters, and inositol phosphates (Turner 2008, Liu et al. 2022). To cope with such situations, a range of beneficial rhizobacteria secrete different phosphatases to dissolve organic phosphorus in soil and utilize the unique phosphorus transport system for uptake and utilization (Fitriatin et al. 2011). The general phosphorus solubilization and uptake system in rhizobacteria consists of four categories of genes, including the phosphorus regulatory transcription factor pho and the TCS phoB/phoR, transport system genes such as pit, pstA, pstB, and ugpQ, the inorganic phosphorus solubilization genes gcd, ppa, and ppx, and organic phosphate mineralization genes such as phoA and phoD (Wu et al. 2022). The phosphorus regulatory transcription regulator pho and the downstream TCS, which are conserved in most bacterial species, are essential in activating phosphorus solubilization and uptake genes in response to a low phosphorus environment. Activation of pho generally induces the expression of a series of downstream reactions to secrete phosphatases and organic acids, therefore mineralizing insoluble organic phosphates (Hulett 1996). In recent years, it has been reported that the constitutive phosphatase (PafA) activity expressed by *Flavobacteria* in the rhizosphere is stronger than that of *Pseudomonas*, which enables *Flavobacteria* to occupy unique phosphorus clearance sites in the rhizosphere and enhance the ability of phosphorus acquisition (Lidbury et al. 2021), making the *Flavobacteria* successful colonizers of the phosphorus solubilizing niche in the rhizosphere.

Iron is an indispensable element that participates in many important biological metabolic processes; in particular, bacterial biofilm formation requires sufficient iron (Qin et al. 2019, Xu et al. 2019a). The total iron in soil is abundant, estimated to be 20–40 g/kg (Bowles 1997); however, most iron is present in insoluble iron oxide precipitates or insoluble high-valence forms. Iron availability is extremely low in neutral and alkaline soils (Moreno-Jiménez et al. 2019). Moreover, plant roots also deploy a strategy that takes up iron and withholds excess iron in vacuoles to restrict pathogen virulence. Therefore, soluble iron is extremely scarce for bacteria in the rhizosphere (Trapet et al. 2021). To increase their competitiveness for iron nutrition in the rhizosphere, most rhizobacteria produce siderophores to chelate ferric iron for colonization in rhizosphere (Stringlis et al. 2018b). Bacterial siderophores can be hijacked by other bacteria to compete for iron (Gu et al. 2020). In addition to competition for soil iron by siderophores, iron competition between rhizobacteria and plants is also a canonical battle field (Xing et al. 2021). It has been recently found that beneficial rhizobacteria also trade with iron resources during bacterial colonization. *Bacillus velezensis* SQR9 deploys the type VII secretion system to export YukE, which inserts into the plant root cell membrane to cause iron leakage to facilitate the iron nutrition and rhizosphere colonization of this strain (Liu et al. 2023).

## Endophyte penetration

Endophytic bacteria colonize the host tissue. Some endophytes can colonize roots from vertical transmission and have been reviewed on vertical transmission (Frank et al. 2017, Guo et al. 2021, Soluch et al. 2021). Here, we focus on the endophytic process after root attachment of the bacteria. The intercellular colonization process has been demonstrated with several model endophytes, such as *Azoarcus* spp., *Paraburkholderia phytofirman*, and *Klebsiella* spp. (Reinhold-Hurek et al. 2007, Turner et al. 2013). The key process is penetration into plant tissue (Hallmann 2001). The infection site selection and the bacterial features involved in lifestyle of root colonization are the key points here.

### Infection site

The infection sites of rhizosphere endophytes are selective. It has been reported that many microorganisms enter plant root tissue by the following three putative pathways: the root tip in the elongation and differentiation zone, the points where lateral roots emerge, and the axils of emerging or developed lateral roots (Reinhold-Hurek and Hurek 1998, James 2000, Mushtaq et al. 2023). James et al. (2002) deployed a GUS-marked strain of the endophyte *Herbaspirillum seropedicae*, a nitrogen-fixing bacterium, to study the rhizosphere colonization site in rice. This bacterium is most abundant on coleoptiles, lateral roots, and at the junctions of the major and lateral roots in the initial step (James et al. 2002, Balsanelli et al. 2010). It enters roots via cracks at the points of lateral root emergence and subsequently colonizes the intercellular spaces of roots (James et al. 2002). Histochemical analysis of seedlings of maize, sorghum, wheat, and rice grown in vermiculite showed that strain *H. seropedicae* LR15 colonized inner tissues. In the early steps of the endophytic association, *H. seropedicae* colonized intercellular spaces of the root cortex; it then occupied the vascular tissue. Colonization was also observed in the external mucilaginous root material at 8 dpi (Roncato-Maccari et al. 2003). *Bacillus megaterium* NCT-2 could penetrate into maize roots through the root tip in the elongation and differentiation zone (Chu et al. 2018). Compant et al. (2005) labeled *Burkholderia* sp. PsJN with GFP and observed the bacterial cells enriched in high numbers at the sites of lateral root emergence. Growing evidence support the idea that the endophytic colonization site is highly restricted by plant, such as by the plant immunity, the suberin, the casparian strip, and some antimicrobial metabolites in root tissues (Philippe et al. 2020, Durr et al. 2021, Fröschel et al. 2021, Kashyap et al. 2022, Verbon et al. 2023).

### Specific features of bacterial endophytes

It seems that the decision of endophytic colonization can be distinct even between bacterial strains with close phylogenetic relationships. For instance, two efficient avocado root tip colonizers, *P. alcaligenes* AVO73 and *P. pseudoalcaligenes* AVO110, display distinct colonization sites; the latter colonizes root wounds and intercellular spaces between root epidermal cells, while the former colonizes only the root surface (Pliego et al. 2008). It is generally agreed that the factors influencing bacterial endophytism are complex and varied. Chen et al. (2020) explored the transcriptome profile of rice upon infection by two endophyte isolates, *Azoarcus olearius* BH72 and *Azospirillum* sp. B510 and found that plants respond quite differently to these two endophytes, suggesting a large variation in molecular interactions during endophytic colonization. But knowledge on the bacterial genetic features that responsible for penetration into root tissue and intercellular lifestyle is still very limited.

Cell wall degradation is expected to be a fundamental skill of endophytic bacteria, even if they do not need to enter the intracellular space. The secretion of cell wall-degrading enzymes, mainly pectinases and cellulases, is known to be involved in bacterial penetration into plant tissue (Compant et al. 2005). A mutant of *A. olearius* BH72 devoid of endoglucanase activity had a decreased ability to colonize rice (Reinhold-Hurek et al. 2006). Rat et al. (2021) tested 197 endophytic bacteria of medicinal plant *Alkanna tinctoria* and found strains expressing cell-wall degrading enzymatic activities might have strong plant growth-promoting activity due to their ability to colonize plant.

A unique respiratory type of metabolism may be essential for an endophyte because the carbon resources and the oxygen in plant tissue are quite different from those in the rhizoplane and soil. For example, the well-studied endophyte *A. olearius* BH72 has a strictly respiratory type of metabolism and cannot utilize common carbohydrates (Krause et al. 2006). A highly adaptive respiratory type can be expected to be essential for root endophytic life of bacteria.

Unique motility may function in evading plant tissue. Böhm et al. (2007) demonstrated that a type IV pili-dependent twitching motility, but not the type-pili itself, mediated the endophyte *A. olearius* BH72 invasion of and establishment inside the plant.

The interaction with plant immunity is expected to be a major trait for the adaptive lifestyle of endophytes. It has been shown that a plant-beneficial endophyte generally elicits a weaker immune response than pathogens. Moreover, Deng et al. (2019) demonstrated that an endophyte *B. subtilis* strain could evade plant defense by producing subtilomycin to mask self-produced flg22. Activation of the immune response or other stress responses is always accompanied by oxidative bursts, which lead to osmotic stress in endophytes, so it can be expected that a successful endophyte also harbors ROS tolerance to address the plant immune response and the ROS produced by plants under stressful conditions. Alquéres et al. (2013) found that the endophyte *G. diazotrophicus* PAL5 showed increased expression of genes encoding ROS-detoxifying enzymes during colonization in rice roots.

In conclusion, knowledge on the molecular mechanism underlying the endophytic lifestyle is still lacking. First, although the feasible and independent solutions for endophyte isolation have been demonstrated, a standardized and unbiased method is urgently needed. A comprehensive genomic comparison will help to determine whether there is a common trait in the genome of bacterial endophytes. To identify genes involved in the endophytic lifestyle rather than contributing to the colonizing process before entering plant tissue using mutational experiments, comparing colonization both on the root surface and in root tissue is necessary. In addition, it could also be that endophytism is transient and opportunistic rather than a strict lifestyle.

## “Cry-for-help” theory for root colonization of rhizobacteria

Several papers demonstrated that stressed plants recruit beneficial bacteria to colonize the root, thereby facilitating the stress-induced opposite effect on plant growth and health (Berendsen et al. 2018, Yuan et al. 2018, Santoyo 2022, Xie et al. 2022, Wen et al. 2023). It is a noteworthy factor that influences bacterial colonization. One of the well-known strategies is the “cry for help” hypothesis, which explains the long-term disease suppressive soil feedback to foliar pathogen attack. The underlying mechanism still remain to be demonstrated (Wang and Song 2022). Although the current understanding of the cross-talk between root exudation, the root immune system, and the “cry for help” response is limited, it can be expected or confirmed that they may be linked internally. Rolfe et al. (2019) proposed three stages for this plant disease-induced long-term response: root immune responses to attackers, stress-induced changes in root exudation of antimicrobials and signaling chemicals, and impacts of root exudates on the rhizosphere microbiome. In addition, evidence has shown that root exudation from abiotic stressed plants also promotes colonization of beneficial rhizobacteria, which function to relieve the stress response of the plant. This stress-induced host selection would highly influence the colonization of beneficial rhizobacteria by changing the immune response and root exudation.

### Biotic stress triggered “cry for help” response

Rudrappa et al. (2008) were the first to provide experimental evidence that aboveground disease alters root exudation of a primary root metabolite, l-malic acid, resulting in increased root colonization by a beneficial rhizobacterial strain. The authors propose that *P. syringae* pathovar *tomato* DC3000 (*Pst* DC3000) infection of *Arabidopsis* leaves induces root secretion of l-malic acid, which acts as a specific signal for chemotaxis and colonization of the biocontrol bacterium *B. subtilis* FB17 in the rhizosphere (Rudrappa et al. 2008). A follow-up study demonstrated that either MAMPs, such as flg22, or the pathogen-derived phytotoxin COR are necessary to induce plants to secrete l-malic acid to promote colonization by *B. subtilis* FB17 (Lakshmanan et al. 2012).

However, the mechanism that triggers the colonization promotion response is unclear. Regulation of the immune system upon perception of foliar pathogens was thought to contribute to influencing root microbiome composition (Lebeis et al. 2015). Foliar attack by pathogens or insects can influence belowground direct and indirect plant defense responses (Bezemer and Van Dam 2005), but the root immune system needs to differentiate between beneficial and pathogenic microbes and mount appropriate, yet diametrically opposed, colonization-enabling or defense responses. However, COR, as a mimic of JA-Ile, was proposed to suppress SA signaling and the flg22-triggered immune response (Li et al. 2005, Melotto et al. 2006), since both flg22 and COR could trigger the colonization promotion response. It is ambiguous how the immune response in aboveground tissue is involved in promoting root colonization by *Bacillus*. It is hypothesized that some defense signaling activated upon infection by pathogen may be positive for beneficial rhizobacterial colonization. Indeed, Yang et al. (2023) found that the SA signaling pathway is essential for eliciting plants to promote root colonization of some biocontrol bacteria for bacterial wilt disease.

Another important case comes from the interaction between *Fusarium* and plants. Liu et al. (2017) used a split-root system to show that inoculation of part of the cucumber root system with *Fusarium* changes numerous root exudates and promotes colonization of the beneficial rhizobacterium *B. velezensis* SQR9 in distal roots, which was linked to increased exudation of tryptophan, a biofilm formation stimulator of strain SQR9. This finding was also corroborated by a comics study by Wen et al. (2023), who found that *Fusarium*-infected cucumber also attracted *Sphingomonas* in addition to *Bacillus* for root colonization by stimulating the genes involved in motility and chemotaxis (Wen et al. 2023). Similarly, Schulz-Bohm et al. (2018) found that upon infection with the fungal pathogen *Fusarium culmorum, Carex arenaria* changed the blend of root-secreted VOCs that promote the colonization of specific bacteria with antifungal properties. Root exudates from *Fusarium*-infected maize also stimulate root colonization of *B. amyloliquefaciens* OR2-30 by stimulating chemotaxis and motility (Xie et al. 2022). In wheat, *Fusarium* infection leads to higher root colonization of *Stenotrophomonas rhizophila* SR80, a dominant beneficial bacterium that induces strong disease resistance by boosting plant defense in aboveground plant parts (Liu et al. 2021).

Upon infection by phytopathogens, plant roots release several antimicrobial compounds, but little is known about their effects on root colonization by beneficial rhizobacteria. One interesting field of how these antimicrobial compounds contribute to the “cry for help” response and affect beneficial bacterial colonization is studies on the rhizosphere function of coumarin. Coumarin is a class of phenolic secondary metabolites synthesized by *Arabidopsis* that can stimulate biofilm formation of *B. subtilis* (Korenblum et al. 2022). Stringlis et al. (2018a) revealed that coumarin scopoletin selectively inhibits the soil-borne fungal pathogens *Fusarium oxysporum* and *Verticillium dahliae*, while growth-promoting and resistance-inducing *Pseudomonas* are highly tolerant to scopoletin. Vismans et al. (2022) found that foliar infection of *Arabidopsis thaliana* by the biotrophic downy mildew pathogen *Hyaloperonospora arabidopsidis* recruits beneficial bacteria that can enhance plant resistance, while it is evident that the coumarin biosynthesis genes MYB72 and F6’H1 in *Arabidopsis* are essential for recruiting beneficial bacterial colonization upon infection. These findings draw the outline of a fascinating “cry for help” response.

### Abiotic stress triggered “cry for help” response

The colonization of beneficial rhizobacteria on roots can also be activated by plants under abiotic stress. For instance, rice during and after drought recruits beneficial *Streptomyces* to colonize the root endosphere (Santos-Medellín et al. 2021). Drought typically decreases the root exudation of plants, but drought-stressed trees have increased root exudation of phenolic acid compounds and quinate to recruit beneficial *Bacillus* and *Pseudomonas* for colonization (Oppenheimer-Shaanan et al. 2022). Root secretion of flavonoids, which is often elevated in plants under abiotic stress, may also be involved in promoting colonization upon stress production. *Arabidopsis* roots under dehydration stress increased flavonoid accumulation within 15 min. The flavonoid naringenin enhances root colonization of *Aeromonas* sp. H1, which is identified as a plant beneficial bacterium capable of enhancing plant resistance to dehydration through transcriptional enhancement of bacterial motility and colonization (He et al. 2022). Hou et al. (2021) demonstrated that *Arabidopsis* under low photosynthesis drives the recruitment of specific rhizobacteria with beneficial effects. Plants under salt stress employ a species-specific strategy to promote colonization by beneficial bacteria in the rhizosphere. Root exudates from the salt-stressed coastal halophyte *Limonium sinense* promote the growth, chemotaxis and finally root colonization of the bacterium *B. flexus* KLBMP 4941 (Li et al. 2021d). An interesting example is coumarins, besides mediating the pathogen-infection-triggered “cry for help” response, coumarins have also demonstrated to be secreted by *A. thaliana* upon iron-deficiency stress to recruit beneficial bacteria (Harbort et al. 2020). Besides the specific molecules, stress-induced plant hormones are essential for plants to recruit beneficial bacteria. Indeed, Chen et al. (2020) found that peanut root could sense the cyanide stress produced by neighboring cassava plants and produce ethylene to recruit beneficial bacteria to adjust to the stressful environment.

## Comparison with pathogenic/symbiotic bacteria for rhizosphere colonization mechanisms

**Figure 2.**
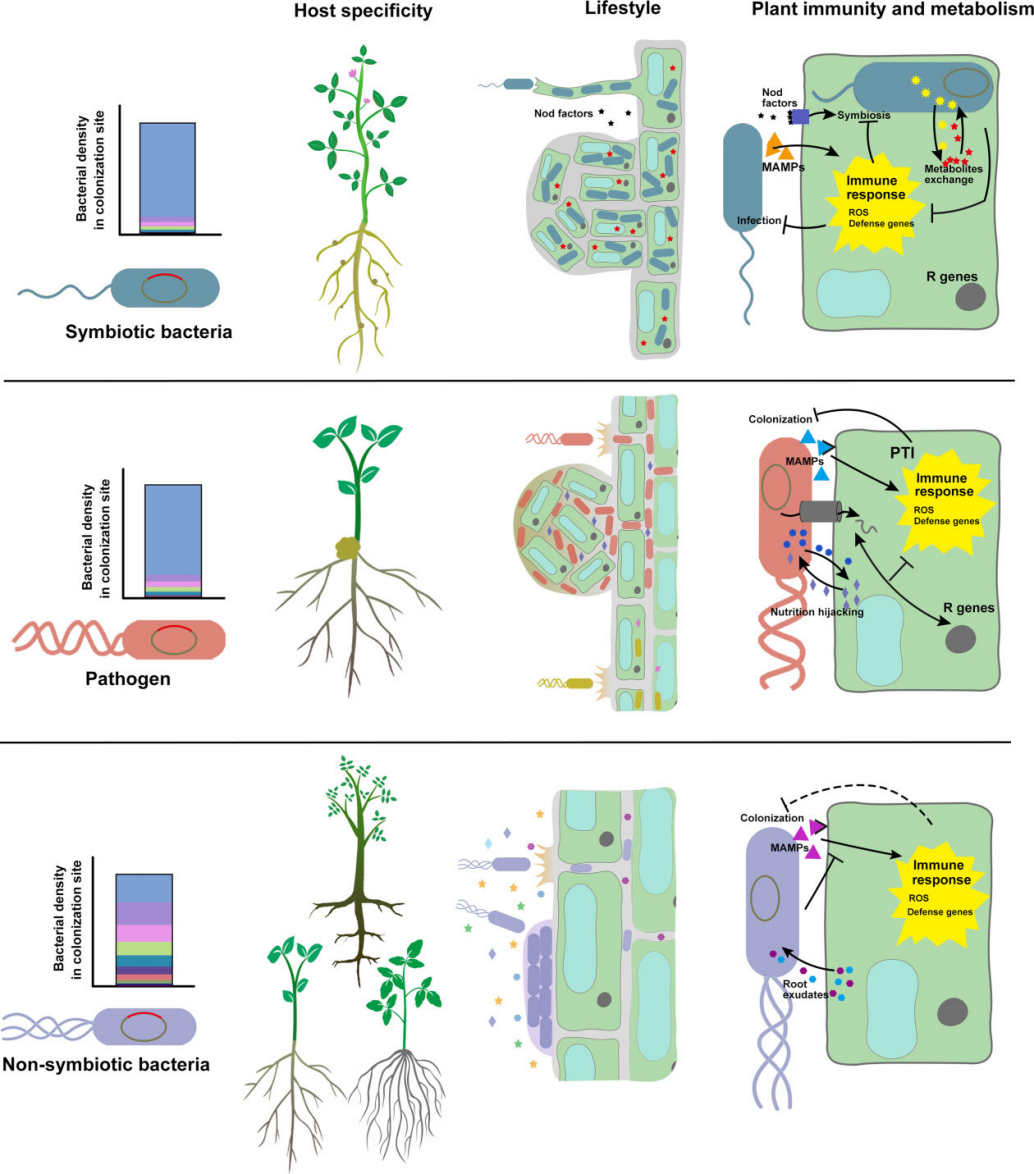
Comparison of the colonization of nonsymbiotic rhizobacteria with symbiotic and pathogenic bacteria. The relative bacterial density of a nonsymbiotic rhizobacterium in its colonization site is lower than that of symbiotic and pathogenic bacteria. Nonsymbiotic rhizobacteria generally have broad host range, while symbiotic and pathogenic bacteria have very specific host. Symbiotic bacteria acquire carbon resources directly from the root cells and feed root cells with nitrogen, pathogenic bacteria hijack plant metabolism and nutrition, while nonsymbiotic rhizobacteria mainly use root exudates and the secretions in intercellular spaces. Symbiotic bacteria have specific interaction with plant immunity to establish infection and symbiosis, pathogenic bacteria block plant immune response by injecting effectors into root cells.

Pathogenic, symbiotic, and nonsymbiotic rhizobacteria represent three groups of root colonizers that are tightly associated with plant. But the comparison of the difference of their colonization mechanisms in rhizosphere is lack. The rhizosphere chemotaxis and root attachment of these bacterial groups are similar, which are mainly by sensing root secreted signals, moving toward rhizosphere, and adhering to root surface, although the signals or cellular molecular pathway involved may different. The colonization process for pathogenic/symbiotic bacteria and the nonsymbiotic beneficial bacteria differed mainly in their specific lifestyles. Most nonsymbiotic rhizobacteria colonize the rhizoplane as a community, some endophytes colonize the intercellular spaces of the root at a controlled low density (Lugtenberg and Kamilova 2009). However, symbiotic bacteria colonize roots intracellularly and sometimes they induce root to develop specific organs, which allow their high populations in root (Tang et al. 2020). Pathogenic bacteria infect root tissues and always grow to a very high density, which is needed for expression of virulence factors (von Bodman et al. 2003). The different lifestyles lead to difference of host specificity, nutrition and metabolism and strategies against plant immunity during colonization in the rhizosphere (Fig. 2).

### Host specificity

Generally, a nonsymbiotic beneficial rhizobacterium can colonize a broad range of host plants. For example, *B. velezensis* SQR9 was isolated from the rhizosphere of cucumber and can colonize *Arabidopsis*, maize and rice efficiently (Liu et al. 2014, Cao et al. 2023a). *Pseudomonas simiae* WCS417 was isolated from the rhizosphere of wheat and induced systemic resistance in *Arabidopsis*, tomato, and many other plant species, suggesting efficient colonization of these plant species (Berendsen et al. 2015). The endophytes *A. olearius* BH72 was isolated from Kallar grass (*Leptochloa fusca* L. Kunth), while it also endophytically colonized rice (Hurek and Reinhold-Hurek 2003). However, relatively strict host selection is observed for symbiotic and pathogenic bacteria. Isolates belonging to *Rhizobiaceae* only infect legumes as a very specific host. One rhizobium strain can not colonize different cultivars from the same host plant species. This opinion is highly supported by the results from Dong et al. (2021), who found that the legume *Medicago truncatula* possesses an SHR–SCR stem cell program in cortical cells to specifically interact with rhizobia for nodulation. Pathogenic bacteria also have strict host selection. For example, one strain from *P. syringae* generally has a very limited host plant species and even a few cultivars from a single plant species, based on which the basis of the pathogenic *P. syringae* can be grouped into pathovars (Xin and He 2013).

The narrow host spectrum for symbiotic and pathogenic bacteria is generally due to their host selection genes, and the presence or absence of these genes determines the infection of a specific host. For example, a common concept of the presence of pathogenic bacteria and symbiotic strains is called avirulent genes, which enable specific nonhost plants to specifically prevent the infection of that strain. These avirulent genes typically mediate immune recognition by nonhost plants (Yang et al. 2010). In contrast, there are currently no reported host selection genes in nonsymbiotic beneficial rhizobacteria. But nonsymbiotic rhizobacteria do have a host preference, which suggest the existence of specific genes determines the colonization of these bacteria (Wippel et al. 2021). Even though, here is currently a tendency to believe that such bacteria use lower amplification rates in association with host plant in exchange for a wider host range.

### Nutrition and metabolism

Lifestyle determines the metabolism of the bacteria. Due to the intracellular life of symbiotic bacteria, their metabolism and carbon resources are largely dependent on their host cells, and therefore, they generally have a more specific metabolites exchange with the host. Intracellular colonization is established and partially controlled by plant genes. For example, rhizobia mainly use the carbohydrates of host plants as carbon resources and feed plants with ammonia during root nodule symbiosis (Yang et al. 2022). Moreover, the respiratory type and redox potential of symbiotic bacteria are highly influenced by the host plant (Yu et al. 2018). Specific metabolism was also observed in the well-studied *Agrobacteria* strategy, during which pathogenic *Agrobacterium* hijacks plant cells by injecting a part of the DNA sequence from the Ti plasmid to produce opines as dedicated carbon resources for *Agrobacterium* itself (Lang et al. 2013, González-Mula et al. 2018, Matveeva and Otten 2021). The plant pathogen *Ralstonia solanacearum* is also able to manipulate plant metabolism to produce GABA to support bacterial nutrition during colonization (Xian et al. 2020).

The nutrition and metabolism of most nonsymbiotic rhizobacteria are not strictly dependent on the host. They mainly use a broad range of organic compounds in root exudates for colonization (Badri and Vivanco 2009). In contrast to the specific carbon resources for bacteria during nodulation or infection, due to the much higher diversity of bacteria than intercellular and intracellular spaces, the bacteria colonizing the root surface should have a broader carbon source utilization spectrum to compete for nutrients in root exudates (Mataigne et al. 2022). The diversity of the bacteria in the rhizosphere led them to share the various compounds of the root exudates (Yang et al. 2017, 2019). Moreover, most nonsymbiotic rhizobacteria can degrade and use the soil-derived carbon resources.

### Plant immunity evading strategy

The lifestyle of pathogenic, symbiotic, and nonsymbiotic bacteria is largely distinctive, leading a quite different strategy to interact with plant immunity. Due to the intracellular lifestyle of symbiotic bacteria, activation of the plant immune response is believed to be harmful to the interaction (Feng et al. 2021b). Most pathogenic bacteria infect root tissue in a high density, eliciting a stressful and PAMP-rich environment; when pathogens do not have immune-blocking strategies, strong PTI and sharply reduced colonization can be expected (Wei et al. 2018). Nonsymbiotic bacteria generally colonize the rhizosphere at a relatively lower density, but ROS accumulation or establishment of immune response within roots has a weaker influence to the colonization of nonsymbiotic bacteria than to the pathogenic and symbiotic bacteria (Buschart et al. 2012, Zhang et al. 2021). This may rely on the different concentrations of antibacterial compounds, such as ROS, in root cells, intercellular spaces, and rhizoplane. The difference has been evident by several studies that blocking the plant immune response evading mechanism in bacteria has a much stronger impact on colonization of rhizobia and pathogenic bacteria than that of nonsymbiotic beneficial bacteria (Liang et al. 2013, Wei et al. 2015, Deng et al. 2019, Pfeilmeier et al. 2019, Yu et al. 2019a, Zhang et al. 2021). To fit their unique lifestyles, pathogenic, symbiotic, and nonsymbiotic bacteria deployed different strategies to evade plant immunity.

Pathogenic and symbiotic bacteria possess highly immunogenic MAMPs. Although many MAMPs from nonsymbiotic rhizobacteria have been identified, current researches suggest those MAMPs elicit a weaker response than that derived from pathogens, which is shown by a lower elicitation of defense gene transcription, a lower oxidative burst, and a higher concentration needed for seedling growth inhibition (Colaianni et al. 2021, Zhang et al. 2021). For example, Colaianni et al. (2021) demonstrated that the flg22 variant from beneficial *Bacillus* can not trigger seedling growth inhibition when applied to a final concentration of 10 nM, a concentration the flg22 variant from *Pst* DC3000 did. However, pathogens use unique secretion system to interfere the PTI therefore establishing disease (Shu et al. 2023). For example, both pathogenic *P. syringae* and *R. solanacearum* deliver effectors into plant cells through the type III secretion system to interfere with the plant immune response for efficient colonization (Yuan et al. 2021, Yu et al. 2022). The nodulation out proteins secreted by symbiotic bacteria have been reported to suppress PTI (Xin et al. 2012). Both symbiotic and pathogenic bacteria show specific interactions with the plant immune system, such as R genes. For rhizobia, it has also been demonstrated that R genes in legumes control the host specificity of rhizobium symbiosis. But different with pathogen, balanced regulation of innate immunity is required for rhizobial infection and symbiosis (Cao et al. 2017, Yang et al. 2022). In contrast, nonsymbiotic rhizobacteria regulate the plant immune response in general as reviewed in the section “Interaction with plant immunity”, rather than through specific interactions as that of pathogenic bacteria and have never been shown to interact with R genes in plants.

## Artificial enhancement of root colonization by beneficial rhizobacteria

**Figure 3.**
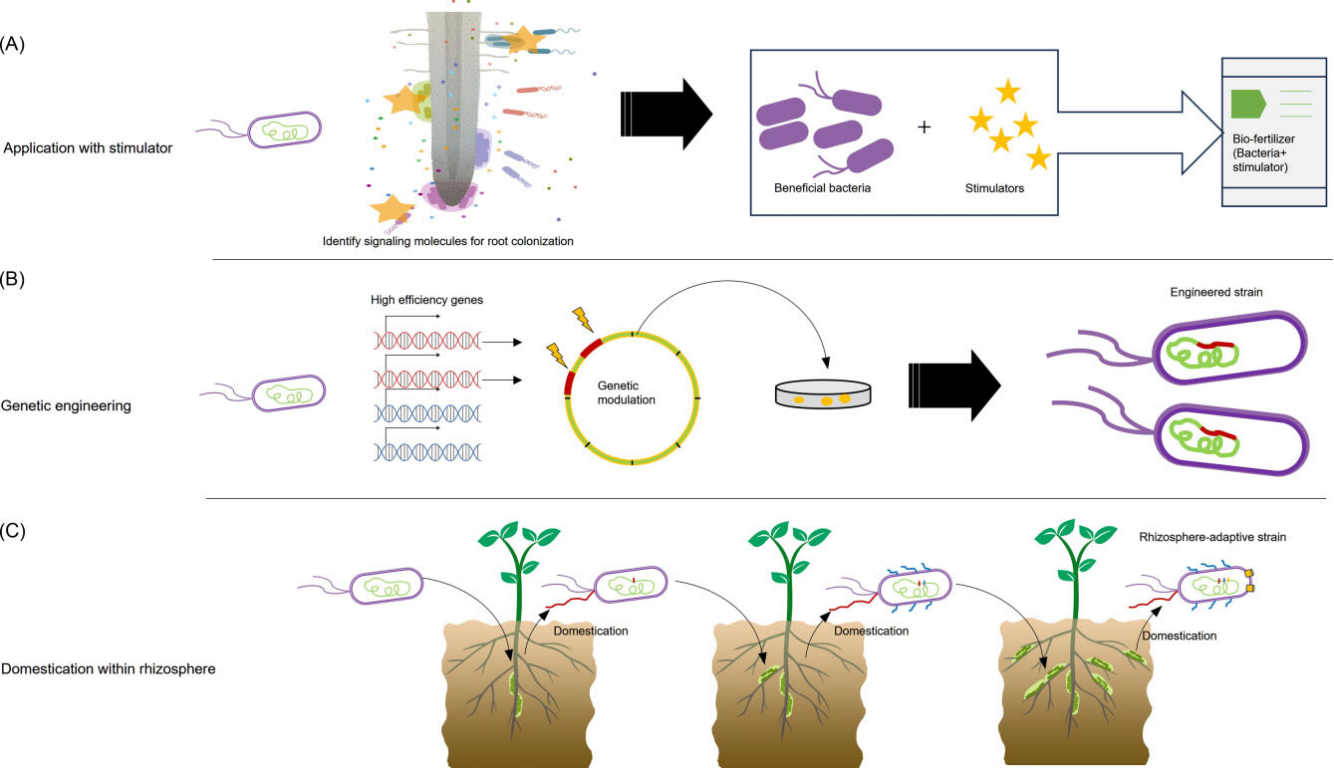
Strategies to promote rhizosphere colonization of nonsymbiotic bacteria. (A) Many compounds in rhizosphere, mainly from the root exudates, have been identified to be positive signaling molecules for beneficial bacterial colonization in rhizosphere. It is a practicable way to develop such molecules as colonization stimulator and applied with the beneficial bacteria together in agriculture. (B) Many bacterial genes have been identified to be positive for rhizosphere colonization with clear mechanisms. Genetic modulation of the beneficial bacteria by introducing “colonization positive” genes would generate engineered strains as better colonizers. (C) Efficient rhizosphere colonization is a beneficial trait for bacteria itself, because rhizosphere supplied more nutrient for bacterial proliferation, therefore a continuous life in rhizosphere is expected to drive the accumulation of “colonization positive” mutations in bacterial genome. So, round-by-round inoculation and reisolation of bacteria in rhizosphere will domesticate an evolved strain as a better colonizer.

The field application of beneficial rhizobacteria is an effective practice for sustainable agriculture, the efficient root colonization of these bacteria is critical for the performance of their beneficial functions. Hence, it is important to develop strategies to enhance the root colonization of beneficial rhizobacteria. This review proposes three strategies, which include the addition of colonization-enhancing substrates, bacterial genetic modulation, and evolution of beneficial rhizobacteria (Fig. 3).

It can be expected that the application of some compounds in root exudates or microbial metabolites may serve as root colonization stimulators given that many studies have demonstrated the role of these compounds in modulating the root colonization of beneficial rhizobacteria. For example, the application of organic acids, such as malic acids, citric acid, and fumaric acid, can enhance root colonization of the beneficial strains *Hansschlegelia zhihuaiae, B. velezensis* SQR9, and *B. pyrrocini* (Zhang et al. 2014, 2015, 2018, 2022, Feng et al. 2018, Han et al. 2023). Therefore, soil amendments can be used to promote beneficial bacterial colonization.

Genetic engineering of beneficial rhizobacteria to respond to specific root exudate compounds is another strategy to enhance colonization. Xu et al. (2019b) developed a xylose-inducible degQ genetically engineered strain of *B. velezensis* SQR9, which can use root secreted xylose as a signal to regulate the phosphorylation level of DegU and then promoted its ability to form biofilm on the root surface. Compared to the wild-type strain, the genetically engineered strain showed greater root colonization ability and biocontrol efficacy in cucumber and tomato (Xu et al. 2019b). Singh et al. (2022b) engineered the beneficial bacterium *A. brasilense* Sp7 with enhanced d-glucose utilization ability and showed significantly increased root colonization in rice compared with the wild-type strain.

One imaginative strategy for improving root colonization ability of beneficial rhizobacteria is coevolution of the strain with the target plant to get the evolved strain, which is termed as targeted domestication. It is known that natural genetic mutations, such as random point mutation and horizontal gene transfer, drive the evolution of bacteria, for example, phage infection drive the evolution of bacterial resistance to phage (Hussain et al. 2021). Therefore, if a bacterial strain was inoculated to the rhizosphere, isolated and reinoculated again, then, after rounds of repeating, the genetic mutations in the evolved bacterial genome that benefit its life in rhizosphere will accumulate due to the survival of the fittest theory. It can be inferred that artificial domestication of bacterial strains within the rhizosphere under monoassociation conditions may accelerate evolution and drive the direction to a better rhizosphere colonizer. Indeed, Blake et al. (2021) found that *B. subtilis* NCIB 3610 differentiated into three different colony morphologies after experimental evolution within the *Arabidopsis* rhizosphere and that a mixture of the three morphotypes colonized the rhizosphere better than each colony alone. Li et al. (2021c) repeatedly inoculated *Pseudomonas protegens* CHA0 in the rhizosphere of *A. thaliana* cultivated in sandy soil for six growth cycles, and they detected 35 mutations within 28 genes in the genome of the evolved isolates. Among them, mutations affecting global regulators, bacterial cell surface structure, and motility accumulated in parallel across multiple evolved strains (Li et al. 2021c). Moreover, the relationship between bacteria and plants has evolved from antagonism to mutualistic cooperation, which is manifested in a stronger ability to utilize rhizosphere exudates and a stronger tolerance to antibacterial substances secreted by plants (Li et al. 2021a). However, the entire trait correlation networks of *P. protegens* CHA0 are recombined after adaptive evolution, showing the loss of stress resistance modules and the linking of those modules that enhance ability after evolution (Li et al. 2021b). Compared with the solid substrate environment, domestication within the *Arabidopsis* rhizosphere under a hydroponic environment places more emphasis on the mobility and recolonization ability of strains (Nordgaard et al. 2022). Rotating croplands provide a more complex ecological environment for bacteria. In an evolutionary experimental study, the evolutionary strains in alternate host environments had a higher degree of parallel evolution at the gene level (Hu et al. 2023). Adaptive mutations in *B. subtilis* NCIB 3610 occurred earlier in the presence of *Pseudomonas* in the rhizosphere, suggesting that a competitive environment accelerates this capacity change (Pomerleau et al. 2023). In conclusion, evolution experiments can be used as an important means to breed beneficial rhizobacteria with improved root colonization and agricultural application. However, this evolution-based domestication is also risky because a slight environmental difference may lead to a butterfly effect on the resultant strains’ features. Moreover, domestication of the bacteria in a simplified environment would weaken the bacterial ability in other environments, such as stress tolerance (Li et al. 2021a, b, c).

## Conclusion and outlook

The importance of rhizobacteria in plant growth, development, and health has been well recognized. Recent studies have revealed many fascinating models that describe complex interactions between rhizobacteria and plant and soil environments. However, compared with the soil-borne pathogenic and symbiotic bacteria of rhizobia, the root colonization of beneficial rhizobacteria has not been comprehensively concluded. Here, we summarized the root colonization of rhizobacteria into several steps. We also compared the difference in the colonization process of those nonsymbiotic beneficial rhizobacteria with symbiotic and pathogenic bacteria. Finally, we discussed the efforts made to improve the root colonization of beneficial rhizobacteria, which will facilitate their agricultural application.

The nonsymbiotic rhizobacteria represent the plant-associated bacteria with the largest abundance and diversity in the rhizosphere. The mechanism of root colonization of nonsymbiotic bacteria is significantly different from that of symbiotic and pathogenic bacteria. The colonization of any nonsymbiotic strain can not reach the abundance level as that of symbiotic or pathogenic bacteria. The symbiotic and pathogenic bacteria colonize the inside root tissues with very high abundance, while most nonsymbiotic beneficial rhizobacteria colonize the root surface or inside root tissue with a low abundance. The differences in colonization site and abundance suggest that the nonsymbiotic rhizobacteria have different root–bacteria interaction mechanisms. In particular, how do plants recognize nonsymbiotic beneficial rhizobacteria and allow colonization?

The rhizosphere environment is rich in other organisms, including fungi, protozoans, viruses, and other bacteria. Moreover, the microbiome in the rhizosphere is dominated by nonplant factors and varies largely depending on environmental factors, such as soil type, temperature, and humidity. Based on these concerns, the root colonization study of beneficial rhizobacteria in more natural conditions and under the holistic view of the rhizosphere microbiome and even the multitrophic interaction level will provide an in-depth understanding of the process and mechanisms in the future. Benefiting from the development of sequencing technology, many studies have made great efforts to use bioinformatic methods to analyze the rhizosphere microbiome.

Finally, the study of rhizobacterial root colonization aims to improve the agricultural application efficiency of biofertilizers, which are mostly isolated from beneficial rhizobacteria. Therefore, our future study of rhizobacterial root colonization should pay more attention to the development of products or biotechnologies based on the process and mechanism understanding to improve the field application effect of beneficial rhizobacteria. More efforts to develop a new generation of biofertilizers that enhance beneficial rhizobacterial colonization should be made to promote the sustainable development of agriculture.
